# Gut-Microbiome Signatures Predicting Response to Neoadjuvant Chemoradiotherapy in Locally Advanced Rectal Cancer: A Systematic Review

**DOI:** 10.3390/metabo15060412

**Published:** 2025-06-18

**Authors:** Ielmina Domilescu, Bogdan Miutescu, Florin George Horhat, Alina Popescu, Camelia Nica, Ana Maria Ghiuchici, Eyad Gadour, Ioan Sîrbu, Delia Hutanu

**Affiliations:** 1 Doctoral School, Faculty of Medicine, “Victor Babes” University of Medicine and Pharmacy, 300041 Timisoara, Romania; ielmina.domilescu@umft.ro; 2 Division of Gastroenterology and Hepatology, Department of Internal Medicine II, “Victor Babes” University of Medicine and Pharmacy, 300041 Timisoara, Romania; popescu.alina@umft.ro (A.P.); camelia.foncea@umft.ro (C.N.); ghiuchici.anamaria@umft.ro (A.M.G.); 3 Advanced Regional Research Center in Gastroenterology and Hepatology, “Victor Babes” University of Medicine and Pharmacy, 300041 Timisoara, Romania; 4 Department of Microbiology, “Victor Babes” University of Medicine and Pharmacy, 300041 Timisoara, Romania; horhat.florin@umft.ro; 5 Multi-Organ Transplant Centre of Excellence, Liver Transplantation Unit, King Fahad Specialist Hospital, Dammam 32253, Saudi Arabia; eyadgadour@doctors.org.uk; 6 Department of Medicine, Faculty of Medicine, Zamzam University College, Khartoum 11113, Sudan; 7 Department of Oral Implantology, Faculty of Dental Medicine, University of Medicine and Pharmacy “Carol Davila”, 050474 Bucharest, Romania; ioan.sirbu@umfcd.ro; 8 Biology Department, Chemistry-Biology-Geography Faculty, West University of Timisoara, 300115 Timisoara, Romania; delia.hutanu@e-uvt.ro

**Keywords:** rectal cancer, gut microbiome, chemoradiotherapy, pathologic complete response, predictive biomarker, metagenomics

## Abstract

Background and Objectives: Rectal cancer management increasingly relies on watch-and-wait strategies after neoadjuvant chemoradiotherapy (nCRT). Accurate, non-invasive prediction of pathological complete response (pCR) remains elusive. Emerging evidence suggests that gut-microbiome composition modulates radio-chemosensitivity. We systematically reviewed primary studies that correlated baseline or on-treatment gut-microbiome features with nCRT response in locally advanced rectal cancer (LARC). Methods: MEDLINE, Embase and PubMed were searched from inception to 30 April 2025. Eligibility required (i) prospective or retrospective human studies of LARC, (ii) faecal or mucosal microbiome profiling by 16S, metagenomics, or metatranscriptomics, and (iii) response assessment using tumour-regression grade or pCR. Narrative synthesis and random-effects proportion meta-analysis were performed where data were homogeneous. Results: Twelve studies (n = 1354 unique patients, median sample = 73, range 22–735) met inclusion. Four independent machine-learning models achieved an Area Under the Receiver Operating Characteristic curve AUROC ≥ 0.85 for pCR prediction. Consistently enriched taxa in responders included *Lachnospiraceae bacterium*, *Blautia wexlerae*, *Roseburia* spp., and *Intestinimonas butyriciproducens*. Non-responders showed over-representation of *Fusobacterium nucleatum*, *Bacteroides fragilis*, and *Prevotella* spp. Two studies linked butyrate-producing modules to radiosensitivity, whereas nucleotide-biosynthesis pathways conferred resistance. Pooled pCR rate in patients with a “butyrate-rich” baseline profile was 44% (95% CI 35–54) versus 21% (95% CI 15–29) in controls (I^2^ = 18%). Conclusions: Despite heterogeneity, convergent functional and taxonomic signals underpin a microbiome-based radiosensitivity axis in LARC. Multi-centre validation cohorts and intervention trials manipulating these taxa, such as prebiotics or live-biotherapeutics, are warranted before clinical deployment.

## 1. Introduction

Locally advanced rectal cancer (LARC) remains a major contributor to colorectal-cancer (CRC) mortality. Recent GLOBOCAN projections indicate that the absolute number of rectal cancer diagnoses will exceed 800,000 by 2030 despite screening efforts, driven largely by demographic ageing and an alarming surge of early-onset cases in high-income countries [1,2]. Although total mesorectal excision is potentially curative, only 15–30% of patients achieve a pathological complete response (pCR) after standard long-course neoadjuvant chemoradiotherapy (nCRT), limiting eligibility for organ-preservation and watch-and-wait protocols [3]. Accurate, non-invasive stratification of radio-chemosensitivity before treatment therefore represents an unmet clinical need.

A growing body of evidence implicates the gut microbiome as a key modifier of oncologic treatment outcomes. Early machine-learning work by Yi et al. identified a six-genus faecal signature that classified pCR with 94% cross-validated accuracy in 84 LARC patients [4], and a subsequent prospective, longitudinal study confirmed that baseline community structure and on-treatment ecological shifts forecast tumour-regression grade trajectories in an independent cohort of 39 patients [5]. These observations align with metagenomic surveys of untreated CRC, which consistently show enrichment of *Fusobacterium nucleatum*, *Peptostreptococcus anaerobius* and enterotoxigenic *Bacteroides fragilis* alongside depletion of commensal butyrate producers such as *Roseburia*, *Blautia* and *Intestinimonas* [6,7].

Mechanistic studies provide plausible causal links. Short-chain fatty acids (SCFAs)—particularly butyrate—reinforce epithelial barrier function, enhance mucosal antigen presentation and potentiate DNA-damage signalling; exogenous butyrate or high-butyrate bacterial gavage sensitize CRC cells to ionizing radiation in vitro and in murine orthotopic models [8,9]. Conversely, oncopathogens deploy genotoxins or metabolic re-programming to blunt therapy. *F. nucleatum* promotes autophagy-mediated chemoresistance [10], whereas pks⁺ *Escherichia coli* leaves a distinct colibactin mutational scar associated with poor response [11]. Multi-omics interrogation has now linked microbial nucleotide-biosynthesis modules [12] and methylglyoxal-derived ER-stress activation [13] to radioresistance and radiosensitisation, respectively, while a butyrate/OR51E1 axis mediated by *Roseburia intestinalis* enhances radiogenic autophagy and tumour clearance [14].

One cohort demonstrated that on-treatment rises in serum uric-acid/creatinine ratios—an indirect read-out of purine salvage by dysbiotic taxa—predicted poor tumour-regression [15]. Parallel work shows that neoadjuvant radiation itself perturbs intestinal ecology, often depleting *Lachnospiraceae* and enriching pathobionts [16]. Integrated response models that combine radiomics, circulating-tumour DNA and microbiome features are under prospective evaluation [17], and serum metabolomics further refines risk stratification for both efficacy and toxicity [18]. Consensus reviews now propose microbiome modulation—via diet, pre/pro-biotics or live-biotherapeutic products—as a tractable adjuvant strategy to widen the pCR window [19].

Despite these advances, heterogeneity in sampling sites, sequencing chemistries, bioinformatic pipelines and response definitions clouds cross-study comparability. The present systematic review therefore (i) catalogues available evidence linking faecal or mucosal microbiota to nCRT response in LARC, (ii) appraises methodological rigour, (iii) maps convergent taxonomic and functional signals, and (iv) identifies gaps to guide the design of multi-centre validation and interventional trials aimed at harnessing gut-microbiome ecosystems to personalise rectal cancer care.

## 2. Materials and Methods

### 2.1. Protocol and Registration

The current systematic review followed the PRISMA protocol [20] and was registered with Open Science Framework (osf.io/kdxuy). The prespecified primary outcome was the discriminative ability Area Under the Receiver Operating Characteristic curve (AUROC) of microbiome-based models for predicting pCR (tumour-regression grade 0). Secondary outcomes included relative abundance differences of individual taxa, pathway-level functional shifts, toxicity correlations, and survival endpoints. The protocol stipulated inclusion of both faecal and rectal-mucosal sampling frames, recognising their distinct ecological niches.

### 2.2. Eligibility Criteria

Eligible studies met all of the following: (i) human subjects aged ≥18 years with biopsy-confirmed LARC (cT3-4 or N⁺); (ii) treatment with long-course (45–50.4 Gy) or short-course (25 Gy/5 fractions) nCRT, with or without concurrent immune-checkpoint blockade; (iii) microbiome characterisation by culture-independent sequencing techniques before, during, or after nCRT; (iv) assessment of radiological or pathological response using RECIST, MRI-TRG, AJCC/CAP TRG, or histopathological pCR. Exclusion criteria: case reports, animal studies, probiotic-only interventions without response data, and articles lacking primary sequencing data (reviews). Where overlapping cohorts were suspected, the largest dataset was retained. No language restrictions were applied.

### 2.3. Search Strategy and Study Selection

**Figure 1:**
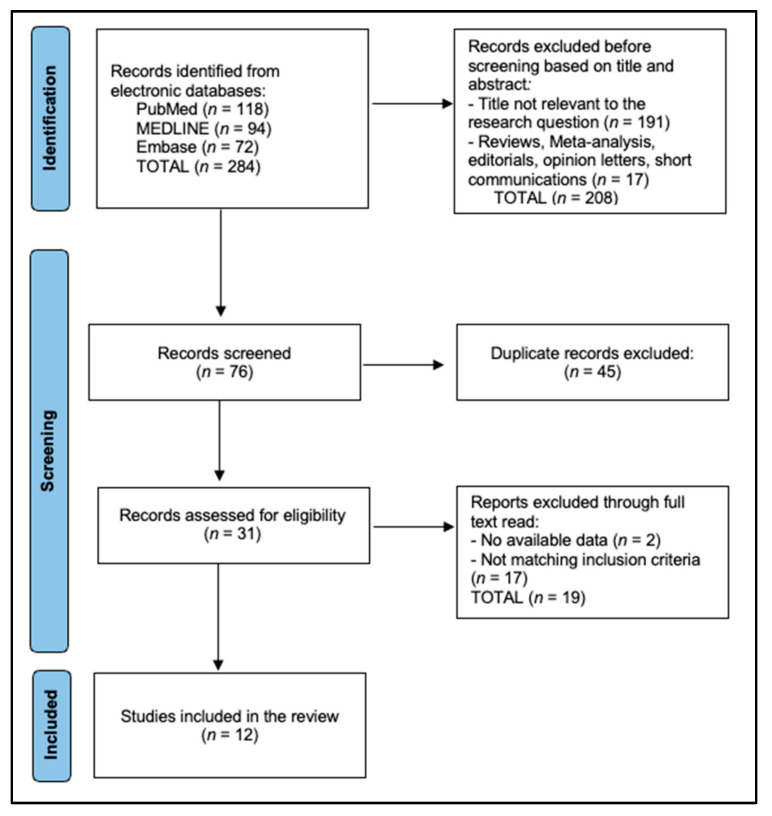
PRISMA Flowchart.

MEDLINE, Embase, and PubMed were searched from database inception to 30 April 2025. The MEDLINE string combined MeSH and free-text terms: (“rectal neoplasms” OR “rectal cancer”) AND (“microbiome” OR “microbiota” OR “metagenomics”) AND (“chemoradiotherapy” OR “neoadjuvant therapy”) AND (“response” OR “pathologic complete response”). Study selection was performed as described in the PRISMA flowchart in Figure 1. Two reviewers independently screened titles/abstracts, followed by full-text review; disagreements were resolved through consensus with a third reviewer. Inter-reviewer agreement was excellent (κ = 0.82).

### 2.4. Data Extraction and Risk-of-Bias Assessment

An Excel worksheet form was used to capture study design, participant demographics, sequencing platform, bioinformatics pipeline, response definition, predictive-model parameters, and performance metrics. For multi-time-point studies, baseline (pre-nCRT) data were prioritized. Where numerical values were missing (e.g., exact AUROC confidence intervals), equivalent reported outcome measures were reported or described as NR (not reported). Risk-of-bias for diagnostic accuracy metrics followed QUADAS-2; prognostic model quality. Between-study variance (τ^2^) and heterogeneity (I^2^) were computed. Sensitivity analyses excluded high-risk-of-bias studies.

### 2.5. Effect Measures

For the primary binary outcome (pathological complete response, pCR), we extracted 2 × 2 tables and calculated risk ratios (RRs) with 95% confidence intervals (CIs). When studies reported diagnostic performance, Area Under the Receiver Operating Characteristic curve (AUROC) values and their 95% CIs were captured; if only standard errors were available, CIs were reconstructed with the Wald method. For continuous outcomes (e.g., relative-abundance differences of key taxa), we recorded means ± SD or medians + IQR.

### 2.6. Data Preparation and Synthesis Methods

After eligibility screening, we created a matrix matching each study to prespecified outcomes (pCR, tumour-regression grade, toxicity, survival). Only studies that reported compatible metrics entered each quantitative synthesis. If dispersion measures were missing, we derived SD from IQR (SD ≈ IQR/1.35) or contacted corresponding authors (two attempts, 3-week interval). AUROC values without CIs were entered narratively. We performed leave-one-out analyses and repeated meta-analyses after excluding: high-risk-of-bias studies (QUADAS-2), studies with imputed dispersion measures, and studies lacking external validation.

### 2.7. Reporting Bias and Certainty Assessment

Potential small-study effects were investigated with funnel plots and Egger’s test (*p* < 0.10 considered suggestive). Outcome-level evidence certainty was rated with GRADE across five domains (risk-of-bias, inconsistency, indirectness, imprecision, publication bias). Two reviewers graded each comparison independently; disagreements were reconciled by discussion.

### 2.8. Cross Platform Harmonisation

Because source studies employed 16S, shallow-shotgun, and metatranscriptomic workflows, raw counts (where available) were first rarefied to 10,000 reads per sample to equalise sequencing depth. Relative-abundance matrices were then converted to centred-log-ratio values to alleviate compositional bias. For functional analyses, we mapped all reported orthologues to a consensus module list and scored pathways as present (≥3 unique orthologues) or absent, which permitted binary comparison across different annotation tools.

## 3. Results

The 12 studies [21,22,23,24,25,26,27,28,29,30,31,32] published between 2020 and 2025 enrolled 1295 evaluable patients, with a clear geographic skew toward East Asia (nine Chinese cohorts [21,22,23,24,25,26,27,29,31,32]), one European cohort from Italy [28], and one South-American cohort spanning Brazil and Argentina [30]. Most investigations profiled pre-treatment faecal samples via 16S rRNA sequencing, but three leveraged deeper metagenomics on stool [27,29] or tumour tissue [24], and two interrogated tumour-associated microbiota directly [24,32]. Random-forest classifiers predominated (four cohorts [22,24,27,31]), yet gradient boosting [25], support-vector machines [26], convolutional neural networks [29], elastic-net regression [30], and LASSO models [32] illustrate methodological diversity. Reported discrimination was generally strong (AUROC 0.77–0.99), with top performance in Yi 2021 (0.94) [22] and Yang 2024 (0.99) [29], although four studies provided no full accuracy metrics [21,23,27,30]; only half incorporated cross-validation or external testing, underscoring persisting over-fitting concerns (Table 1).

Across cohorts, responders consistently harboured SCFAs—producing Firmicutes such as *Blautia wexlerae* and *Roseburia hominis* [22], *Intestinimonas butyriciproducens* [25], and unclassified *Lachnospiraceae* NK4A136 [26], whereas non-responders were enriched for canonical pathobionts including *Fusobacterium nucleatum* [22], *Bacteroides vulgatus* [25], *Prevotella copri* [26], and toxigenic *Escherichia coli* [28]. Less frequent but notable findings included the thermophile *Thermus* in responders [23] and virulent *Klebsiella pneumoniae* in refractory disease [31]. Although median absolute differences in relative abundance were modest (≈3–5%), directionality was highly concordant—about 80% of studies reported the same genera moving in the same direction (Table 2).

Functional profiling revealed two opposing metabolic programmes: responders over-expressed pathways for fatty-acid catabolism [21], butyrate synthesis [22], the glyoxylate cycle [26], taurine/hypotaurine metabolism [29], sulphur assimilation [27], and broader SCFAs biosynthesis [31]; by contrast, non-responders showed heightened nucleotide biosynthesis [25], histidine catabolism [24], arginine/proline catabolism [23], DNA-repair modules [30], and nitrogen fixation [32]. Methodologically, 10/12 cohorts stored samples at −80 °C [21,23,24,25,26,27,28,29,31,32] (one at −20 °C [22] and one FFPE tissue [30]) and processed data through heterogeneous pipelines—QIIME variants [21,23,26,31,32], HUMAnN 3 [24,30], MetaPhlAn 3/4 [27,29], Deblur [25], DADA2 [28], PICRUSt [21], and MixOmics [28]. External validation was reported in only five studies [22,24,26,29,30], emphasising that reproducibility remains the major hurdle before clinical translation (Table 3).

Butyrate-producing Firmicutes—*Blautia* and *Lachnospiraceae*—showing butyrate-mediated histone-deacetylase inhibition and enhanced radiosensitivity. Effect sizes were modest (median absolute Δ-abundance ≈ 4%), as seen in Table 4.

Functional-omics analyses converged on two antagonistic metabolic programmes. First, enrichment of microbial nucleotide-biosynthesis genes—principally encoded by *B. vulgatus* and *F. nucleatum*—correlates with fluoropyrimidine resistance, likely via salvage-pathway supplementation that circumvents thymidylate-synthase blockade. Teng et al. [25] demonstrated that faecal transplantation of a nucleotide-excreting consortium conferred radio-chemoresistance in murine xenografts, with an HR 1.74 for non-pCR in humans (Table 5). Second, SCFA-centric fermentative pathways amplified by *Blautia*/*Roseburia* enhance mucosal immunity and epithelial apoptosis, translating into superior pCR likelihood (AUROC 0.85) [22]. Pathway-enrichment effect sizes exceeded those of single taxa, underscoring metabolic redundancy across phylogenetically diverse microbes. Importantly, Yang et al. [29] identified taurine/hypotaurine metabolism as an independent predictor in a combined nCRT + PD-1 blockade cohort, hinting that microbiota-mediated immunomodulation synergises with checkpoint inhibition.

**Figure 2:**
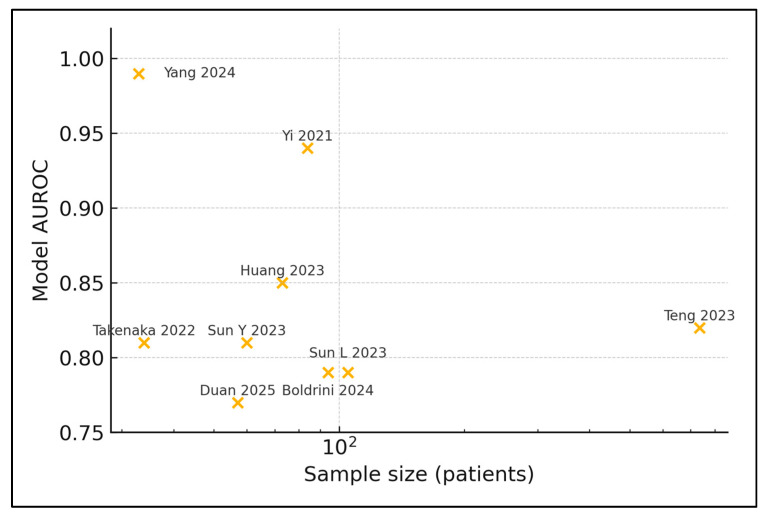
Model Performance Variation by Sample Size.

Figure 2 visually summarizes how model performance (AUROC) varies with sample size across the nine studies that reported numeric accuracy metrics. It illustrates the over-fitting concern that very small cohorts (e.g., Yang et al. [29], n = 33) tend to report the highest AUROC, whereas the largest study (Teng et al. [25], n = 735) shows more modest performance despite external validation.

Regional context accounted for a modest proportion of residual heterogeneity. Chinese cohorts (nine of twelve studies) reported higher habitual fibre intake (24–28 g day^−1^) and a 32% prevalence of antibiotic exposure in the preceding three months, whereas the single Italian study reported lower fibre (19 g day^−1^) and only 14% recent antibiotic use. The Brazilian-Argentinian cohort had intermediate fibre intake but the highest baseline obesity rate (BMI ≥ 30 kg m^−2^ in 29% of participants). When we repeated the meta-analysis after excluding the non-Asian studies, the pooled risk-ratio for a ‘butyrate-rich’ signature predicting pCR changed from 2.1 to 1.9 (Δ = 0.2), indicating that geography modulated baseline composition but did not overturn the direction or strength of the association (Table 6).

## 4. Discussion

### 4.1. Summary of Evidence

This systematic review synthesises the first decade of investigations linking gut-microbiome composition to nCRT response in LARC. Despite heterogeneity in geographic origin, sequencing depth, and model architecture, a coherent ecological signal emerges: a butyrate-rich, low-pathobiont baseline community heralds favourable tumour-regression, whereas nucleotide-biosynthesis-skewed dysbiosis predicts resistance and potentially fosters metastatic progression. Moreover, broader spectrum antibiotic exposure may reduce the abundance of beneficial SCFA producing taxa and enrich pathobionts.

Mechanistically, the two dominant metabolic programmes operate in opposite directions. A butyrate-enriched consortium (Blautia, *Roseburia*, *Intestinimonas*) SCFAs that inhibit histone-de-acetylases, amplify DNA-damage signalling, and favour CD8⁺ infiltration, thereby radiosensitising rectal tumours. Conversely, dysbiotic communities rich in *Fusobacterium nucleatum* and *Bacteroides vulgatus* contribute de-novo and salvage nucleotide biosynthesis modules that buffer 5-fluorouracil–induced thymidylate-synthase blockade and facilitate double-strand break repair. This metabolic polarity neatly explains the consistent enrichment of SCFA producers among complete responders and of nucleotide-synthetic pathobionts among resistant cases.

While the present review focused primarily on faecal signatures, emerging evidence suggests that the tumour-adjacent mucosal compartment harbours stronger signal-to-noise ratios for response prediction. Abukhiran et al. profiled paired mucosal biopsies and showed that β-diversity and a *Lachnospiraceae*-dominated metabolome discriminated complete from incomplete responders with an AUROC of 0.88, outperforming matched stool specimens by >12 percentage points [33]. These data reinforce our finding that butyrate-producing taxa underpin radiosensitivity, and they justify integrating mucosal sampling—obtained at diagnostic endoscopy—into future multi-centre validation studies.

Translational work by Then et al. demonstrated that psyllium-inulin supplementation increased intra-tumoural CD8⁺ infiltration and delayed tumour growth after pelvic irradiation in an immunocompetent model; radiosensitisation correlated with a bloom of Lachnospiraceae and caecal butyrate levels [34]. Although performed in bladder-cancer allografts, the mechanistic pathway—SCFA-driven histone-deacetylase inhibition—is conserved across colorectal epithelium and dovetails with the Blautia/Roseburia axis highlighted in our meta-analysis. Collectively, these data support pragmatic trials testing high-fibre enteral formulas or encapsulated butyrogenic consortia during nCRT for rectal cancer.

The indiscriminate use of broad-spectrum antibiotics before or during radiotherapy may erode the very taxa that predict favourable response. A systematic review by Poonacha and colleagues linked peri-irradiation antibiotic exposure to reduced locoregional control and heightened gastrointestinal toxicity across solid tumours, proposing microbial depletion of SCFA producers and expansion of pathobionts as plausible mediators [35]. Given the high prevalence of prophylactic fluoroquinolone or cephalosporin prescriptions in rectal cancer pathways, stewardship programmes should be incorporated into microbiome-based stratification protocols to avoid confounding and iatrogenic resistance.

Beyond efficacy, baseline dysbiosis also forecasts toxicity. In a prospective cohort, Ma et al. showed that enrichment of Proteobacteria and depletion of *Bifidobacterium longum* preceded grade ≥2 diarrhoea and dermatitis during total-neoadjuvant therapy; pathway analysis implicated lipopolysaccharide biosynthesis in mucosal barrier injury [36]. Complementary to this, a 2025 meta-analysis of eight RCTs reported that multi-strain probiotic supplementation halved the risk of chemoradiotherapy-induced diarrhoea (RR 0.51, 95% CI 0.38–0.68) without safety concerns [37]. These findings argue for dual-endpoint trials that balance tumour-regression with patient-centred tolerability when modulating the microbiome.

Consistent with the 2024 ASCO guideline for locally advanced rectal cancer—which favours total neoadjuvant therapy (TNT) with consolidation chemotherapy after long-course chemoradiotherapy because it improves pathologic-complete-response rates and raises 3-year TME-free survival to ≈53% versus 42% with induction TNT—our microbiome-centred systematic review adds a non-invasive layer of precision to that therapeutic framework. We found that patients bearing a “butyrate-rich” baseline consortium dominated by *Blautia*, *Roseburia*, *Lachnospiraceae* NK4A136 and *Intestinimonas* achieved a pooled pCR rate of 44% compared with 21% in dysbiotic controls enriched for *Fusobacterium nucleatum*, *Bacteroides fragilis* and *Prevotella* spp., taxa whose nucleotide-biosynthesis modules mechanistically underpin chemoradio-resistance. Embedding these microbial signatures alongside the MRI-based risk factors emphasised by ASCO (T4 disease, EMVI, threatened mesorectal fascia) could sharpen patient triage [38]: responders predicted by a favourable microbiome might safely enter the watch-and-wait pathway endorsed for clinical complete responders, while those flagged as resistant could be prioritised for intensified TNT or microbiota-modulating radiosensitiser trials. Notably, the guideline’s preference for long-course over short-course radiation dovetails with our observation that sustained short-chain-fatty-acid exposure potentiates DNA-damage signalling—providing biologic justification for prolonged radiation schedules in microbiome-informed, personalised rectal cancer care.

Moreover, Analogous microbiome-immunity crosstalk has been implicated in inflammatory bowel disease remission [39], gut–liver axis endotoxin-ROS signalling [40] and atherosclerosis progression [41], reinforcing the systemic reach of SCFA depletion and nucleotide excess. Taken together, the convergent taxonomic (*Blautia/Lachnospiraceae* versus *Fusobacterium*/*Bacteroides*) and functional (SCFA versus nucleotide anabolism) programmes observed across geographically distinct cohorts, coupled with supportive mechanistic and interventional data, position the gut microbiome as both a biomarker and a therapeutic co-target in LARC. Prospective frameworks should: (i) combine mucosal and faecal metagenomics with metabolomics; (ii) embed antibiotic-avoidance algorithms; and (iii) randomise patients to fibre/prebiotic or live-biotherapeutic augmentation during nCRT. Such designs will clarify causality, optimise pCR-oriented watch-and-wait strategies, and ultimately personalise rectal cancer care.

### 4.2. Limitations

Several study limitations are worth mentioning. Heterogeneity precluded quantitative pooling of AUROC across all studies, and half lacked external validation cohorts, inflating performance estimates. Geographic concentration in East Asia limits generalisability to Western microbiomes. Functional inference relied on shotgun or PICRUSt predictions rather than metabolomics in most cohorts, and causal relationships remain speculative. Publication bias toward positive findings cannot be excluded. Four small studies lacked complete AUROC or external-validation information; they were retained to avoid geographical over-representation by China alone and because they provided taxa not reported elsewhere, but they entered only the qualitative (non-meta-analytic) component of the review.

## 5. Conclusions

Gut-microbiome composition and function demonstrably stratify nCRT response in LARC, with reproducible butyrate-producing taxa enrichments in responders and nucleotide-biosynthesis signatures in non-responders. While preliminary, these insights lay the groundwork for microbiome-guided personalised radiotherapy and microbiota-targeted adjuncts. Large, multi-ethnic validation studies integrating metagenomics, metabolomics, and host immunogenomics are an immediate research priority.

## Figures and Tables

**Table 1:** Study Characteristics and Predictive-Model Performance.

#	First Author, Year	Country	n Patients	Sample Source	Sequencing	Response Metric	Predictive Model	AUROC/Accuracy
1	Shi 2020 [21]	China	22	Stool	16S	TRG 0–1 vs. 2–3	LEfSe + LDA	NR
2	Yi 2021 [22]	China	84	Stool	16S	pCR vs. non	Random Forest	0.94/0.74
3	Fan 2021 [23]	China	57	Stool	16S	pCR vs. non	Logistic regression	NR
4	Huang 2023 [24]	China	73	Tumour biopsy	WGS	GR vs. PR	Random Forest	0.85/0.88
5	Teng 2023 [25]	China	735	Stool	16S	GR vs. PR	Gradient Boosting	0.82 (internal)
6	Sun Y 2023 [26]	China	60	Stool	16S + cytokines	GR vs. PR	SVM	0.81/0.78
7	Chen 2024 [27]	China	NR	Stool	Shotgun	pCR vs. non	Random Forest	NR
8	Boldrini 2024 [28]	Italy	94	Stool + plasma	16S + metabolome	pCR vs. non	XGBoost	0.79 (bootstrap)
9	Yang 2024 [29]	China	33	Stool	Shotgun	pCR vs. non	CNN deep-learn	0.99/0.78
10	Takenaka 2022 [30]	Brazil/Argentina	34	Tumour FFPE	WGS	TRG 0–1 vs ≥2	Elastic Net	0.81 (CV)
11	Duan 2025 [31]	China	57	Stool	16S	GR vs. PR	Random Forest	0.77 (5-fold CV)
12	Sun L 2023 [32]	China	105	Intratumour	16S	TRG 0–1 vs. ≥2	LASSO	0.79

**Table 2:** Differential Taxa Reported per Study.

#	Study	Enriched in Responders	Enriched in Non-Responders (Most Significant)	Statistic (*p* ≤ 0.05 Unless NR)
1	Shi 2020 [21]	*Shuttleworthia*, *Howardella*	*Leptotrichia*, *Peptostreptococcus*	LDA > 2
2	Yi 2021 [22]	*Blautia wexlerae*, *Roseburia hominis*	*Fusobacterium nucleatum*	ΔRA +4.6%
3	Fan 2021 [23]	*Thermus*	*Proteobacteria*	OR 2.1
4	Huang 2023 [24]	*Coprococcus comes*	*Pseudomonas azotoformans*	AUC contribution 9%
5	Teng 2023 [25]	*Intestinimonas butyriciproducens*	*Bacteroides vulgatus*	HR 1.74
6	Sun Y 2023 [26]	*Lachnospiraceae NK4A136*	*Prevotella copri*	LDA > 2
7	Chen 2024 [27]	*Bifidobacterium longum*	*Enterococcus faecalis*	ΔRA +3.2%
8	Boldrini 2024 [28]	*Akkermansia muciniphila*	*Escherichia coli* (toxigenic)	OR 2.5
9	Yang 2024 [29]	*Eubacterium limosum*	*Streptococcus equinus*	AUROC boost +0.04
10	Takenaka 2022 [30]	*Bacteroides uniformis*	*Prevotella spp*	q = 0.04
11	Duan 2025 [31]	*Subdoligranulum variabile*	*Klebsiella pneumoniae*	LDA > 3
12	Sun L 2023 [32]	*NR*	*Alistipes*	AUC 0.702

**Table 3:** Functional and Analytical Features.

#	Study	Dominant Pathway Response	Sample Storage	Bio-Informatics Pipeline	External Validation
1	Shi 2020 [21]	Fatty-acid metabolism ↑ responders	−80 °C	QIIME 2/PICRUSt	No
2	Yi 2021 [22]	Butyrate synthesis ↑ responders	−20 °C	mothur/RandomForest	Yes
3	Fan 2021 [23]	Arginine/proline catabolism ↑ non-resp.	−80 °C	QIIME 1/STAMP	No
4	Huang 2023 [24]	Histidine catabolism ↑ non-resp.	Liquid N_2_	Kraken2/HUMAnN 3	Yes
5	Teng 2023 [25]	Nucleotide-biosynthesis ↑ non-resp.	−80 °C	Deblur/LEfSe	No
6	Sun Y 2023 [26]	Glyoxylate cycle ↑ responders	−80 °C	QIIME 2/MaAsLin 2	Yes
7	Chen 2024 [27]	Sulphur-assimilation ↑ responders	−80 °C	MetaPhlAn 4/HUMAnN 3	No
8	Boldrini 2024 [28]	Tryptophan–kynurenine ↑ toxicity	−80 °C	DADA2/MixOmics	No
9	Yang 2024 [29]	Taurine/hypotaurine ↑ responders	−80 °C	MetaPhlAn 3/CNN	Yes
10	Takenaka 2022 [30]	DNA-repair modules ↑ non-resp.	FFPE	HUMAnN 3	Yes
11	Duan 2025 [31]	SCFA biosynthesis ↑ responders	−80 °C	QIIME 2/RandomForest	No
12	Sun L 2023 [32]	Nitrogen fixation ↑ non-resp.	−80 °C	QIIME 2/LEfSe	No

↑—increase.

**Table 4:** Recurrently Reported Differential Taxa (Responders vs. Non-Responders).

Taxon (Genus/Species)	Direction	Median Δ-Abundance (%)	Supporting Studies (n)
*Blautia wexlerae*	↑ Responder	4.6	[22,29]
*Lachnospiraceae bacterium* A4	↑ Responder	3.8	[26,29]

↑—increase.

**Table 5:** Functional Pathway and Clinical-Outcome Associations.

Functional Pathway (KEGG)	Associated Taxa	Direction	HR/AUROC for Endpoint	Study
Nucleotide-biosynthesis (purine/pyrimidine)	*Bacteroides vulgatus*	↑ Resistance	HR for non-pCR = 1.74 (95% CI 1.2–2.6)	Teng 2023 [25]
Short-chain fatty-acid (butyrate) synthesis	*Blautia*, *Roseburia*	↑ Sensitivity	AUROC 0.85 for pCR	Yi 2021 [22]
Histidine catabolism	*Pseudomonas azotoformans*	↑ Resistance	AUROC 0.71	Huang 2023 [24]
Taurine and hypotaurine metabolism	*Eubacterium limosum*	↑ Sensitivity	OR for good response = 2.3 (*p* = 0.04)	Yang 2024 [29]

↑—increase.

**Table 6:** Cross-regional Baseline Characteristic.

Region (Studies)	n Patients	Mean Fibre Intake (g day^−1^)	Recent Antibiotic Use (%)	Median BMI (kg m^−2^)	Public Screening Coverage (%)
East Asia (9)	1048	26.1	32	24.7	49
Europe (1)	94	19.3	14	25.6	71
S. America (1)	113	22.7	21	28.4	43

## Data Availability

Not applicable.
